# Cathepsin L Plays a Role in Quinolinic Acid-Induced NF-Κb Activation and Excitotoxicity in Rat Striatal Neurons

**DOI:** 10.1371/journal.pone.0075702

**Published:** 2013-09-20

**Authors:** Yan-Ru Wang, Shu Qin, Rong Han, Jun-Chao Wu, Zhong-Qin Liang, Zheng-Hong Qin, Yan Wang

**Affiliations:** Department of Pharmacology and Laboratory of Aging and Nervous Diseases (SZS0703), Soochow University School of Pharmaceutical Science, Wen Jing Road, Suzhou, China; The University of Texas MD Anderson Cancer Center, United States of America

## Abstract

The present study seeks to investigate the role of cathepsin L in glutamate receptor-induced transcription factor nuclear factor-kappa B (NF-κB) activation and excitotoxicity in rats striatal neurons. Stereotaxic administration of the N-methyl-d-aspartate (NMDA) receptor agonist Quinolinic acid (QA) into the unilateral striatum was used to produce the *in vivo* excitotoxic model. Co-administration of QA and the cathepsin L inhibitor Z-FF-FMK or 1-Naphthalenesulfonyl-IW-CHO (NaphthaCHO) was used to assess the contribution of cathepsin L to QA-induced striatal neuron death. Western blot analysis and cathepsin L activity assay were used to assess the changes in the levels of cathepsin L after QA treatment. Western blot analysis was used to assess the changes in the protein levels of inhibitor of NF-κB alpha isoform (IκB-α) and phospho-IκB alpha (p-IκBα) after QA treatment. Immunohistochemical analysis was used to detect the effects of Z-FF-FMK or NaphthaCHO on QA-induced NF-κB. Western blot analysis was used to detect the effects of Z-FF-FMK or NaphthaCHO on QA-induced IκB-α phosphorylation and degradation, changes in the levels of IKKα, p-IKKα, TP53, caspase-3, beclin1, p62, and LC3II/LC3I. The results show that QA-induced loss of striatal neurons were strongly inhibited by Z-FF-FMK or NaphthaCHO. QA-induced degradation of IκB-α, NF-κB nuclear translocation, up-regulation of NF-κB responsive gene TP53, and activation of caspase-3 was strongly inhibited by Z-FF-FMK or NaphthaCHO. QA-induced increases in beclin 1, LC3II/LC3I, and down-regulation of p62 were reduced by Z-FF-FMK or NaphthaCHO. These results suggest that cathepsin L is involved in glutamate receptor-induced NF-κB activation. Cathepsin L inhibitors have neuroprotective effects by inhibiting glutamate receptor-induced IκB-α degradation and NF-κB activation.

## Introduction

Dysfunction of glutamate receptors is observed in some neurological diseases, including Alzheimer's disease, Parkinson's disease, and schizophrenia [1], [2]. Glutamate receptors have several members and the NMDA receptor is one of them [2]. NMDA receptor channels have several unique features [1]. Studies have shown that they are involved in different physiological processes including acute and chronic neurological disorders, psychiatric disorders, and neuropathic pain syndromes [3]. In primary rat neurons, downregulation of NMDA receptors can inhibit the toxicity induced by glutamate [4].

Quinolinic acid (QA) is an NMDA agonist. When it is administered to laboratory animals, it can cause neurotoxic effects that mimic certain neurodegenerative diseases [5]. Excitotoxicity may play a key role in some central nervous system diseases and is considered to be a major mechanism of cell death [6], [7]. The nuclear translocation factor nuclear factor-kappa B (NF-κB) due to IκB-α degradation is involved in excitotoxicity, which is induced by NMDA and non-NMDA receptor agonists [8]. Our recent studies have also demonstrated that QA activates apoptosis and autophagy, evidenced by increases in the expression of pro-apoptotic proteins, such as TP53, PUMA and Bax, and autophagy regulatory proteins, such as DRAM1, LC3II/LC3I, and beclin 1 [9].

Autophagy is a tightly regulated, cell self-eating process. Increased numbers of autophagosomes and autolysosomes are, under certain conditions, considered to be a prominent ultrastructural feature of degenerating or dying neurons [10]. Autophagy is associated with various neuropathological conditions [11]. Our recent studies have demonstrated that autophagy/lysosomal pathway played important roles in excitotoxic neuronal injury [12], [13]. Cathepsin L is first found in lysosomes as a degrading protease [14]–[16], involved in lysosomal protein degradation [17]. It is a member of the papain superfamily of cysteine proteases and exists in many cells [18], [19]. Furthermore, cathepsin L is found in secretory vesicles of rat pituitary GH4C1 [20] and mouse NIH3T3 cell lines [21]. Cathepsin L is implicated in neuropeptide production in secretory vesicles [22]. Additionally, cathepsin L contributes to a variety of pathological processes, such as cancer and neurodegeneration [23]–[25]. Upregulation of the expression of cathepsin L is detected, and it is considered to be a hallmark, in both cancer and progeria [26]. In AD models, lysosomal hydrolase was released from lysosomes because of the loss of lysosomal membrane impermeability [27]. In 6-OHDA-induced model of PD, the immunoreactivities of cathepsin L increase in the substantia nigra [28]. Furthermore, in human neuroblastoma SH-SY5Y cells, cathepsin L plays a role in 6-OHDA-induced apoptosis and Parkinsonian neurodegeneration [29].

Our previous studies suggested that NF-κB pathway contributed to glutamate receptor-mediated excitotoxicity [13], [30]. We speculate that cathepsin L may play a role in excitotoxicity-induced activation of NF-κB. The present study investigates the effects of cathepsin L inhibitors on QA-induced IκB-α degradation, NF-κB activation, and excitotoxic neuronal death. The results suggest that cathepsin L inhibitors have neuroprotective effects by inhibiting glutamate receptor-induced degradation of IκB-α, NF-κB nuclear translocation, and excitotoxic injury.

## Materials and Methods

### 1. Animals

Sprague-Dawley rats (250–280 g) were obtained from the Experimental Animal Center of Soochow University (certificate No 20020008, Grade II). All the Sprague-Dawley rats used in the experiments were maintained in climate controlled, pathogen-free conditions with a 12-/12-h light/dark cycle. The animals received water and food *ad libitum*. Care and handling of these animals were approved by the Institutional Animal Care and Use Committee of Soochow University and were in accordance with the national guidelines for laboratory animal care.

### 2. Stereotaxic drug administration

Rats were anesthetized with 4% chloral hydrate (400 mg·kg-1). A Kopf stereotaxic apparatus, as described by Qin *et al.* (1996), was used to perform stereotaxic drug administration. QA was injected in the unilateral striatum.

Rats (n = 6 in each group) were infused with QA (60 nmol, 1 µL, infused at 0.4 µL·min^−1^) or vehicle (normal saline, 1 µL), then killed 6, 12 and 24 h later under 4% chloral hydrate anesthesia in order to study the time-course of QA-induced alterations of IκB-α, p-IκBα, and lysosomal enzymes. Striata were dissected for Western blot analysis and cathepsin L activity assay.

Rats were treated with intrastriatal infusion of the cathepsin L inhibitor Z-FF-FMK (2.5, 5, 10 nmol), NaphthaCHO (2.5, 5, 10 nmol), or vehicle (DMSO, 1 µL) 10 min prior to intrastriatal injection of QA (60 nmol) in order to determine the contributions of cathepsin L to QA-induced death of striatal neurons. Z-FF-FMK is an irreversible inhibitor, whereas NaphthaCHO is a reversible inhibitor of cathepsin L. Fourteen days after treatment, rats (n = 6 in each group) were killed and brain sections (40 µm) were cut with a cryostat for Nissl staining and unbiased stereology cell counting.

Rats were treated with intrastriatal infusion of Z-FF-FMK (5 nmol) or NaphthaCHO (5 nmol) 10 min prior to intrastriatal injection of QA (60 nmol), and then were killed 12 h later for immunohistochemical analysis in order to evaluate the effects of Z-FF-FMK and NaphthaCHO on QA-induced NF-κB activation.

Rats were treated with intrastriatal infusion of Z-FF-FMK (5 nmol) or NaphthaCHO (5 nmol) 10 min prior to intrastriatal injection of QA (60 nmol), and then were killed 12 h later in order to evaluate the effects of Z-FF-FMK and NaphthaCHO on QA-induced IκB-α phosphorylation and degradation, and changes in the levels of IKKα, p-IKKα, TP53, caspase-3, beclin1, p62 and LC3II/LC3I. Striata were dissected for Western blot analysis.

### 3. Nissl staining and stereology cell counting

The classical Nissl staining procedure using Cresyl violet was described previously (Paxinos & Watson, 1998). Brain sections (40 µm coronal sections) were immersed for 5 min in each of the following: xylene twice; 100% alcohol twice; 95% alcohol; and 70% alcohol. Brain sections were dipped in distilled water and stained with 0.5% Cresyl violet for 30 min. Stained sections were washed in water for 5 min, and then dehydrated through 70, 95 and 100% alcohol. Sections were then cleared in xylene and cover-slipped. Sections were examined with an inverted fluorescence microscope (Eclipse TE2000U, Nikon, Tokyo, Japan). A CCD camera was used to capture digital images, which were exported to Sigma Scan Pro 5. A clear margin of neuron degeneration and gliosis could be identified and used for assay of lesion size. Lesion size was expressed as percent of the total size of each striatum. Sections at the levels of 1.4 mm and 0.6 mm anterior to the Bregma, and 0.2 mm posterior to the Bregma were used for quantitative analyses of lesion size and neuronal number (Paxinos & Watson, 1998). Twelve brain sections were used for cell counting for the striatum of each animal, with the interval of every fourteenth consecutive brain section. Cell counting was done with Optical Fractionator microscopy. The total number of neurons in the striatum was then statistically analyzed with the software, Stereo Investigator (MBF BioScience, Williston, VT, USA). The neuronal numbers were converted to percentage of control (vehicle-treated striatum) and expressed as mean ± SEM.

### 4. Western blot analysis

Western blot analysis was performed as described by Qin *et al.* (1999). Striatal tissue samples were homogenized in Western blot lysis buffer containing (in mM): Tris-HCl at pH 7.4 (10), NaCl (150), 1% Triton X (100), EDTA (5), phenylmethylsulfonyl fluoride (1), as well as 1% sodium deoxycholate, 0.1% sodium dodecyl sulfate (SDS), 280 u·L^−1^ aprotinin, 50 mg·L^−1^ leupeptin, benzamidine, and 7 mg·L^−1^ pepstain A. The homogenate was then centrifuged at 10600×g for 10 min at 4°C, and supernatant was preserved at −70°C for later use. A BCA kit was used to determine protein concentration. Sixty micrograms of protein from each sample was subject to electrophoresis on 10% SDS-polyacrylamide electrophoresis gel using a constant current. Proteins were transferred to nitrocellulose membranes and incubated in Tris-buffered saline containing 0.2% Tween-20 (TBST) and 3% non-fat dry milk for 3 h with: (a) mouse monoclonal anti-cathepsin L antibody, (b) rabbit polyclonal anti-IκBα antibody, (c) mouse monoclonal anti-phospho-IκBα antibody, (d) rabbit polyclonal anti-IKKα antibody, (e) rabbit polyclonal anti-p-IKKα antibody, (f) mouse monoclonal anti-TP53 antibody, (g) rabbit polyclonal anti-cleaved caspase-3 antibody, (h) rabbit polyclonal anti-beclin 1 antibody, (i) rabbit polyclonal anti-LC3 antibody, or (j) rabbit polyclonal anti-P62 antibody. Membranes were washed and incubated with horseradish peroxidase-conjugated second antibody (anti-mouse or anti-rabbit) in TBST containing 3% non-fat dry milk for 1 h. SuperSignal West Pico Chemiluminescent Substrate (Thermo Scientific, Rockford, IL, USA), was used to detect immunoreactivity. The signal intensity of primary antibody binding was quantitatively analyzed with Sigma Scan Pro 5 and was normalized to a loading control β-actin. The specificity of these antibodies has been tested and reported in the data sheets provided by vendors.

### 5. Immunofluorescence

Rats were anesthetized with 4% chloral hydrate and transcardially perfused with phosphate-buffered saline (PBS; pH 7.4) followed by PBS containing 4% paraformaldehyde (pH 7.4). Perfusion-fixed brains were post-fixed in PBS containing 4% paraformaldehyde overnight. Coronal brain sections (40 µm thick) were cut with a cryostat, washed in PBS for 3×10 min, and blocked in PBS containing 1% normal bovine serum albumin and 0.1% Triton X-100 for 1 h at room temperature. Free-floating sections were incubated with rabbit polyclonal anti-NF-κB p65 antibody in the above-mentioned blocking solution at 4°C for 48 h in order to examine the cellular location of NF-κB p65. Sections were washed three times with PBS and incubated with fluorescent Alexa 488 anti-rabbit secondary antibodies. After 1 h incubation and several rinses, sections were incubated with DAPI for 10 min. After rinses, sections were mounted on glass slides and cover-slipped with Vectorshield fluorescent mounting medium. A Nikon C1 laser-scanning confocal unit (Nikon D-Eclipse C1) attached to an inverted microscope (Nikon Eclipse TE2000-E, Tokyo, Japan) was used to collect images.

### 6. Cathepsin L activity assay

Cathepsin L activity assay was performed with a fluorescence-based assay kit (Abcam) according to the manufacturer's instructions. Each striatal tissue sample was homogenized in 700 µl cell lysis buffer, and centrifuged for 10 min at 10,000×g. Supernatant was transferred to a fresh tube, and kept on ice. Protein concentrations were determined using a BCA kit (Pierce, Rockford, IL). The assay was performed by addition of 100 µg of cell lysate to a 96-well plate and 50 µl of Reaction Buffer was added to each sample. Then 2 µl of the 10 mM Ac-FR-AFC substrate (200 µM final concentration) was added to each assay sample and incubated at 37 °C for 1–2 h. A fluorometer equipped with a 400-nm excitation filter and 505-nm emission filter (TECAN, INFINITE M1000 PRO) was used to read samples.

### 7. Data analysis and statistical procedures

All data are expressed as mean ± SEM. Data were subjected to one-way ANOVA using the GraphPad Prism software statistical package (GraphPad Software, San Diego, CA, USA) in order to establish significance. When a significant group effect was found, post-hoc comparisons were performed using the Bonferroni t-test to examine special group differences. Independent group t-tests were used for comparing two means. The criterion for significance was set at *P*≤0.05.

### 8. Drugs, chemicals reagents and other materials

QA (Sigma, St Louis, MO, USA), Z-FF-FMK and NaphthaCHO (Calbiochem, San Diego, CA, USA), BCA kit (Pierce, Bedford, MA, USA), mouse monoclonal anti-Cathepsin L antibody and rabbit polyclonal anti-LC3 antibody (Abcam, Cambridge, MA, USA), rabbit polyclonal anti-IκBα antibody (Millipore, Billerica, MA, USA), mouse monoclonal anti-Phospho-IκBα antibody (Cell Signaling, Woburn, MA, USA), rabbit polyclonal anti-IKKα antibody (Cell Signaling, Woburn, MA, USA), rabbit polyclonal anti-p-IKKα antibody (Cell Signaling, Woburn, MA, USA), mouse monoclonal anti-TP53 antibody (Cell Signaling, Woburn, MA, USA), rabbit polyclonal anti-cleaved caspase-3 antibody and rabbit polyclonal anti-NF-κB p65 antibody (Cell Signaling, Woburn, MA, USA), rabbit polyclonal anti-beclin 1 antibody (Santa Cruz Biotechnology, CA, USA), rabbit polyclonal anti-P62 antibody (Enzo life science, Exeter, UK), β-actin (Sigma), Fluorescent Alexa 488 anti-rabbit secondary antibody (Molecular Probes, Eugene, OR, USA), DAPI (Sigma), Vectorshield fluorescent mounting medium (Vector Labs, Burlingame, CA, USA), Cathepsin L activity assay kit (Abcam, Cambridge, MA, USA).

## Results

### 1. Contribution of cathepsin L to QA-induced cell death

**Figure 1:**
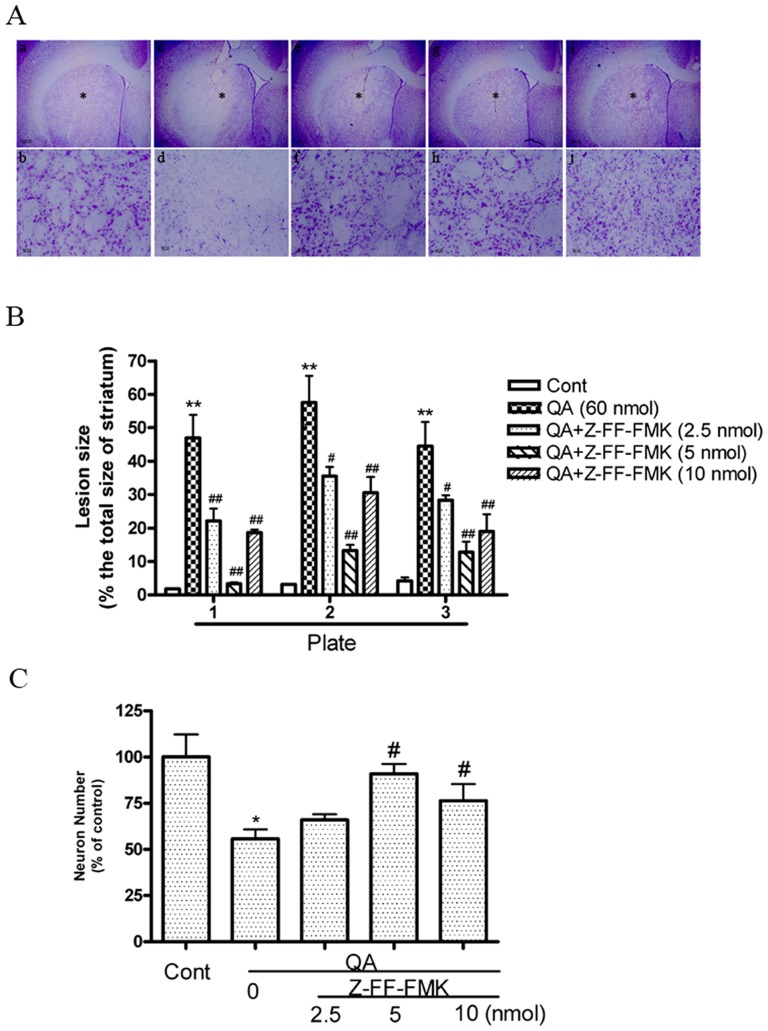
The effects of the cathepsin L inhibitor Z-FF-FMK on QA-induced striatal damage. Rats were treated with intrastriatal injection of Z-FF-FMK (2.5, 5, 10 nmol), 10 min prior to QA (60 nmol) injection. Rats were killed 14 days after QA treatment. Paraformaldehyde-fixed brain sections were stained with Nissl. **A:** The effects of Z-FF-FMK on QA-induced striatal damage. Representative micrographs were taken in the center of drug injection (adjacent to needle tracks). **a and b:** Vehicle. **c and d:** QA. **e and f:** QA+Z-FF-FMK (2.5 nmol). **g and h:** QA+Z-FF-FMK (5 nmol). **i and j:** QA+Z-FF-FMK (10 nmol). b, d, f, h and j (×200) were enlarged from areas indicated with asterisks in a, c, e, g and i (×20). Scale bar  = 200 µm in a, c, e, g and i; scale bar  = 20 µm in b, d, f, h and j. **B:** Quantitative analysis of the effects of Z-FF-FMK (2.5, 5, 10 nmol) on lesion size. Three Nissl-stained sections from each animal were used for quantitative analysis of lesion size caused by QA. Plate 1: sections taken at 1.4 mm anterior to the Bregma. Plate 2: sections taken at 0.6 mm anterior to the Bregma. Plate 3: sections taken at 0.2 mm posterior to the Bregma (The Stereotaxic Atlas of The Rat Brain by Xin-Min Bao, Si-Yun Shu; People's Health Press). Striatal images were captured and exported to Sigma Scan Pro 5 for determining lesion size. Lesion size was expressed as percent of the total size of the striatum of each section. Bars represent mean ± SEM, n = 6 animals per group. Statistical comparisons were carried out with one-way ANOVA followed by Bonferroni *t* test. **C:** The effects of Z-FF-FMK on QA-induced loss of striatal neurons. 12 Nissl-stained sections (with the interval of every 14 successive brain sections) were used for counting neuronal numbers with an Optical Fractionator microscope and stereology software. The neuronal number of the total striatum was expressed as percent of control (vehicle-treated group). Bars represent mean ± SEM, n = 6 animals per group. Statistical comparisons were carried out with one-way ANOVA followed by Bonferroni *t* test. The difference was not statistically significant between QA and QA+Z-FF-FMK (2.5 nmol) treatment. * *P*<0.05 *vs.* control group; ** *P*<0.01 *vs.* control group; # *P*<0.05 *vs.* QA-treated group; ## *P*<0.01 *vs.* QA-treated group.

**Figure 2:**
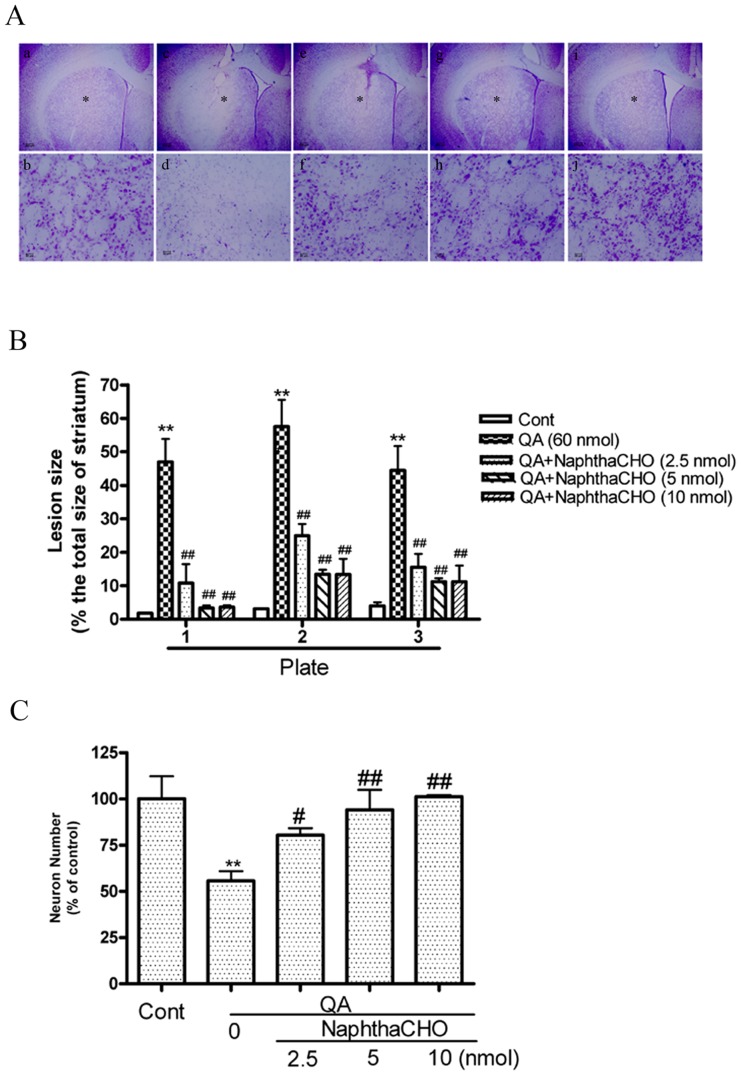
The effects of the cathepsin L inhibitor NaphthaCHO on QA-induced striatal damage. Rats were treated with intrastriatal injection of NaphthaCHO (2.5, 5, 10 nmol), 10 min prior to QA (60 nmol) injection. Rats were killed 14 days after QA treatment. Paraformaldehyde-fixed brain sections were stained with Nissl. **A:** The effects of NaphthaCHO on QA-induced striatal damage. Brain sections were photographed with a microscopy equipped with a CCD camera. Representative micrographs were taken in the center of drug injection (adjacent to needle tracks). **a and b:** Vehicle. **c and d:** QA. **e and f:** QA+ NaphthaCHO (2.5 nmol). **g and h:** QA+ NaphthaCHO (5 nmol). **i and j:** QA+ NaphthaCHO (10 nmol). b, d, f, h and j (×200) were enlarged from areas indicated with asterisks in a, c, e, g and i (×20). Scale bar  = 200 µm in (a, c, e, g and i);  = 20 µm in (b, d, f, h and j). **B:** Quantitative analysis of the effects of NaphthaCHO (2.5, 5, 10 nmol) on lesion size. Three Nissl-stained sections from each animal were used for quantitative analysis of lesion size caused by QA. Plate 1: sections taken at 1.4 mm anterior to the Bregma. Plate 2: sections taken at 0.6 mm anterior to the Bregma. Plate 3: sections taken at 0.2 mm posterior to the Bregma (The Stereotaxic Atlas of The Rat Brain by Xin-Min Bao, Si-Yun Shu; People's Health Press). Striatal images were captured and exported to Sigma Scan Pro 5 for determining lesion size. Lesion size was expressed as percent of the total size of the striatum of each section. Bars represent mean ± SEM, n = 6 animals per group. Statistical comparisons were carried out with one-way ANOVA followed by Bonferroni *t* test. **C:** The effects of NaphthaCHO on QA-induced loss of striatal neurons. 12 Nissl-stained sections (with the interval of every 14 successive brain sections) were used for counting neuronal numbers with an Optical Fractionator microscope and stereology software. The neuronal number of the total striatum was expressed as percent of control (vehicle-treated group). Bars represent mean ± SEM, n = 6 animals per group. Statistical comparisons were carried out with one-way ANOVA followed by Bonferroni *t* test. ** *P*<0.01 *vs.* control group; # *P*<0.05 *vs.* QA-treated group; ## *P*<0.01 *vs.* QA-treated group.

The role of lysosomes and lysosomal enzymes, including cathepsins and some lipid hydrolases, in programmed cell death associated with apoptotic or autophagic phenotypes was demonstrated in cultured cells and living animals [31]. The present study assessed the cumulative loss of striatal neurons after 14 days of QA intoxication in order to examine if cathepsin L is involved in QA-induced neuronal death. The results have shown that QA injection caused profound neuronal loss in rat striatum (Figure 1A and Figure 2A). The lesion size was measured in three coronal sections from each striatum (Plate 1: 1.4 mm anterior to the Bregma; Plate 2: 0.6 mm anterior to the Bregma; Plate 3: 0.2 mm posterior to the Bregma). Cathepsin L inhibitor Z-FF-FMK or NaphthaCHO significantly reduced lesion size induced by QA (Figure 1B and Figure 2B). Twelve sections from each animal were used for determining the loss of striatal neurons with unbiased stereology technology (Figure 1C and Figure 2C). Consistently, the results have shown that the number of striatal neurons was significantly higher in rats pretreated with Z-FF-FMK or NaphthaCHO than that in rats treated with QA only. However, the present study shows that neuroprotection offered by Z-FF-FMK at 10 nmol was not as efficient as at 5 nmol. We observed that 10 nmol Z-FF-FMK itself caused minor striatal damage (Figure S1). In addition, we have tested the effects of cathepsin B inhibitor Ac-LVK-CHO on QA-induced cell death with Nissl staining assay. The results have shown that the cathepsin B inhibitor Ac-LVK-CHO also significantly reduced QA-induced striatal cell death (Figure S2).

### 2. Cathepsin L contributed to QA-induced NF-κB activation

**Figure 3:**
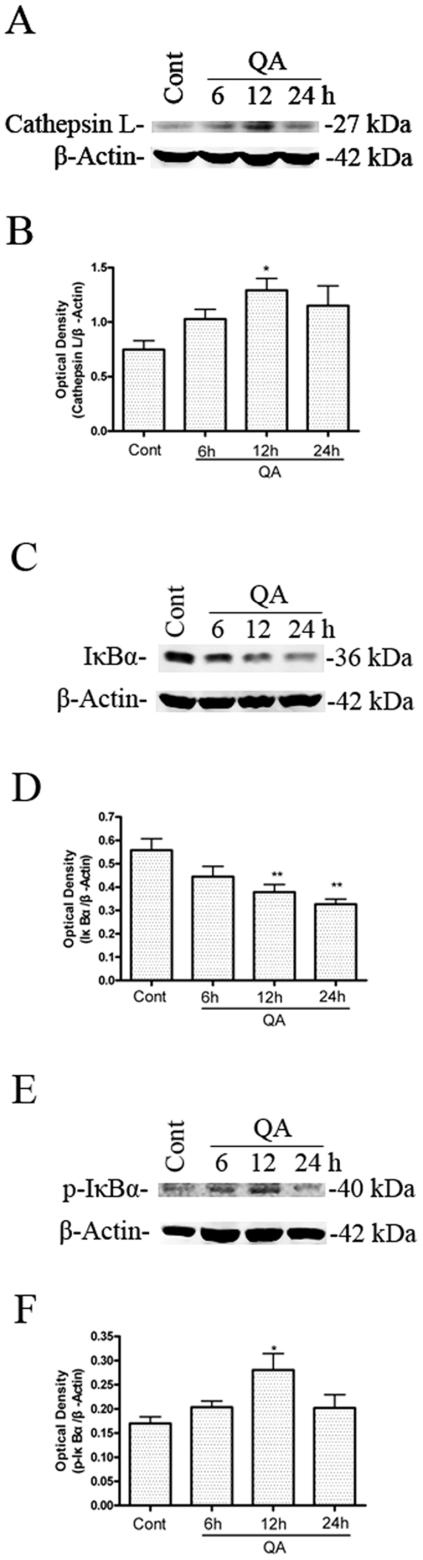
The effects of QA treatment on protein expression levels of cathepsin L, IκB-α and p-IκBα. Rats were treated with intrastriatal injection of QA (60 nmol) and killed 6, 12 and 24 h after drug administration. Striatal tissues were dissected for preparation of striatal extracts for immunoblotting. Optical densities of respective protein bands were analyzed with Sigma Scan Pro 5 and normalized with loading control (β-actin). Data are expressed as Mean ± SEM (n = 6). Statistical comparisons were carried out with one-way ANOVA followed by Bonferroni *t*-test. * *P*<0.05 *vs.* control group; ** *P*<0.01 *vs.* control group.

The time-course of QA-induced changes in cathepsin L protein levels in rat striatum were evaluated at time points 6, 12, and 24 h after infusion of QA. A significant increase in protein levels of cathepsin L was observed 12 h after intrastriatal infusion of QA (Figure 3A and 3B).

**Figure 4:**
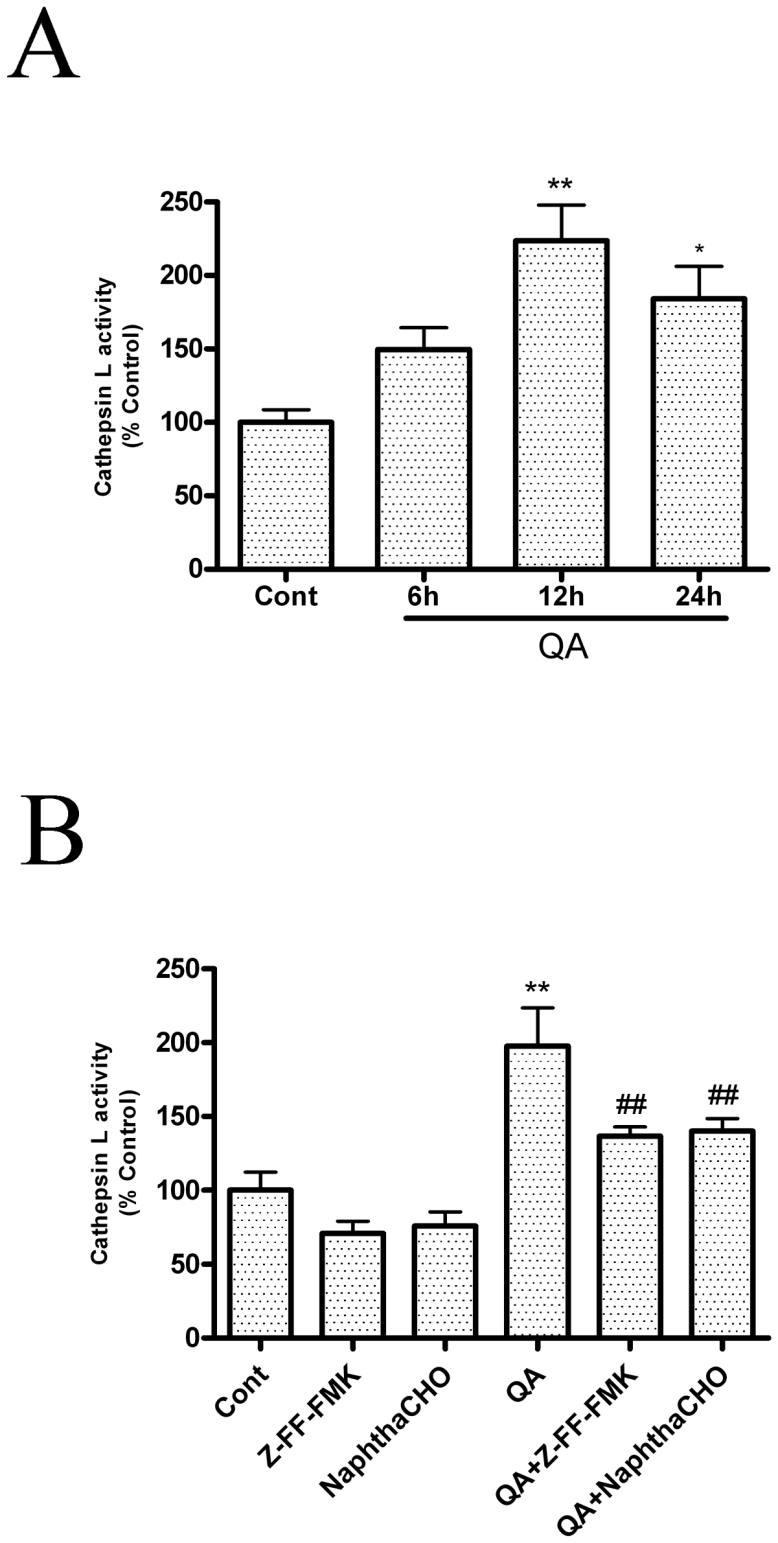
Activation of cathepsin L after QA treatment. A: The time-course of QA-induced increases in cathepsin L activity. Animals were treated as described in the legend to Figure 3. Cathepsin L activity in striatal lysates was determined with a fluorescence-based assay. **B: Effects of Z-FF-FMK and NaphthaCHO on cathepsin L activation.** Rats were pretreated with intrastriatal injection of Z-FF-FMK (5 nmol) or NaphthaCHO (5 nmol) 10 min prior to intrastriatal injection of QA (60 nmol) and were killed 12 h later. Control animals received vehicle injection only. Striata were dissected for assay of cathepsin L activity using a fluorescence-based assay kit. The results were expressed as percent of control (vehicle injection) after performing statistical analysis. Bars represent Mean ± SEM (N = 6). Statistical comparisons were carried out with one-way ANOVA followed by Bonferroni *t*-test. * *P*<0.05 vs. control group; ** *P*<0.01 vs. control group; ## *P*<0.01 vs. QA-treated group.

Cathepsin L activity was determined after QA treatment in order to further analyze QA-induced upregulation of cathepsin L. The time-course of QA-induced changes in cathepsin L activity in rat striatum was evaluated at time points 6, 12, and 24 h after infusion of QA. QA markedly increased cathepsin L activity in rat striatum 12 h and 24 h after QA administration (Figure 4A). The increase in cathepsin L activity induced by QA was significantly inhibited by pretreatment with Z-FF-FMK or NaphthaCHO (Figure 4B). Z-FF-FMK had no effects on protein levels or activity of cathepsin B in the present experimental conditions (Figure S3).

**Figure 5:**
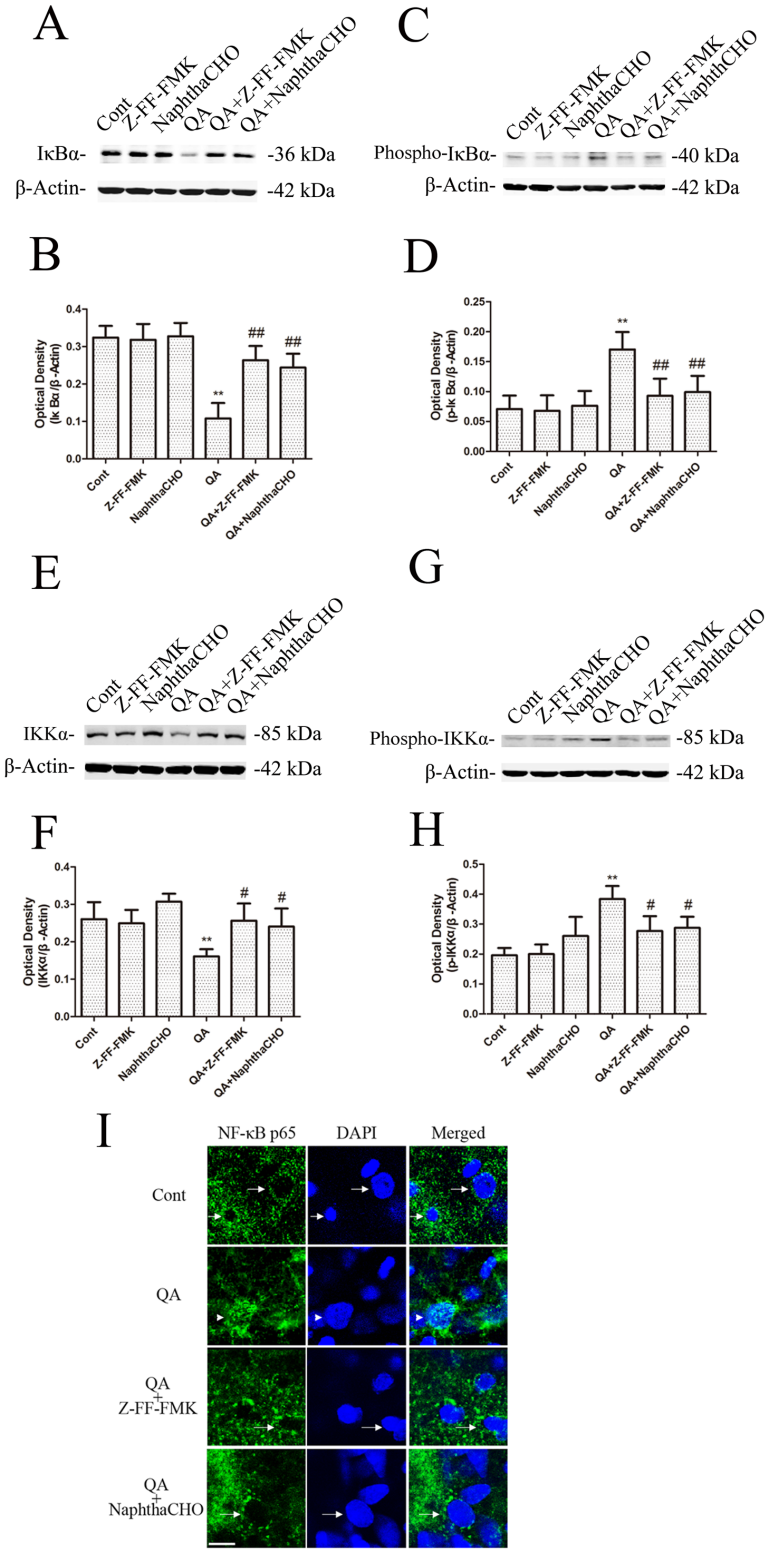
The effects of Z-FF-FMK and NaphthaCHO on QA-induced changes of IκB-α, p-IκB-α, IKKα and p-IKKα protein levels and cellular localization of NF-κB p65. Rats were pretreated with intrastriatal injection of Z-FF-FMK (5 nmol) or NaphthaCHO (5 nmol) 10 min prior to intrastriatal injection of QA (60 nmol) and were killed 12 h later. Control animals received vehicle injection only. A, B, C, D, E, F, G, H: Striatal tissues were dissected for preparation of striatal extracts for immunoblotting. Optical densities of respective protein bands were analyzed with Sigma Scan Pro 5 and normalized with loading control (β-actin). Data are expressed as Mean ± SEM (n = 6). One-way ANOVA followed by Bonferroni *t*-test was used to carry out statistical comparisons. * *P*<0.05 *vs.* control group; ** *P*<0.01 *vs.* control group; # *P*<0.05 *vs.* QA-treated group; ## *P*<0.01 *vs.* QA-treated group. I: Brain sections were processed for immunofluorescence of NF-κB p65. A confocal microscope was used to examine brain sections. Note: NF-κB p65 expression was located in the cytoplasm in the control striatum (arrows). NF-κB p65 nuclear translocation was seen 12 h after QA administration (arrowheads). Pretreatment with the Cathepsin L inhibitor (Z-FF-FMK or NaphthaCHO) reduced QA-induced nuclear translocation of NF-κB p65. Scale bar  = 10 µm.

Our previous studies found that QA- or KA-induced neuronal apoptosis involved activation of NF-κB and induction of TP53 proapoptotic proteins [32]–[34]. In this study, we also found that IκB-α protein levels in rat striatum decreased (Figure 3C and 3D) and p-IκBα protein levels increased (Figure 3E and 3F) after infusion of QA. The effects of Z-FF-FMK or NaphthaCHO on protein levels of IκB-α, p-IκBα, IKKα and p-IKKα were assessed to determine if cathepsin L inhibitors blocked activation of NF-κB,. Z-FF-FMK and NaphthaCHO inhibited QA-induced phosphorylation and degradation of IκBα (Figure 5A, 5B, 5E and 5F). Meanwhile Z-FF-FMK and NaphthaCHO also attenuated QA-induced p-IKKα induction (Figure 5C, 5D, 5G and 5H).

The cellular localization of NF-κB p65 was examined in the present study to further evaluate if cathepsin L plays a role on QA-induced activation of NF-κB. In the control striatum, NF-κB p65 expression was located mainly in the cytoplasm. NF-κB nuclear translocation was seen 12 h after QA administration. Pretreatment with the cathepsin L inhibitor Z-FF-FMK or NaphthaCHO reduced QA-induced nuclear translocation of NF-κB (Figure 5I).

### 3. Cathepsin L was involved in apoptotic and autophagic processes

**Figure 6:**
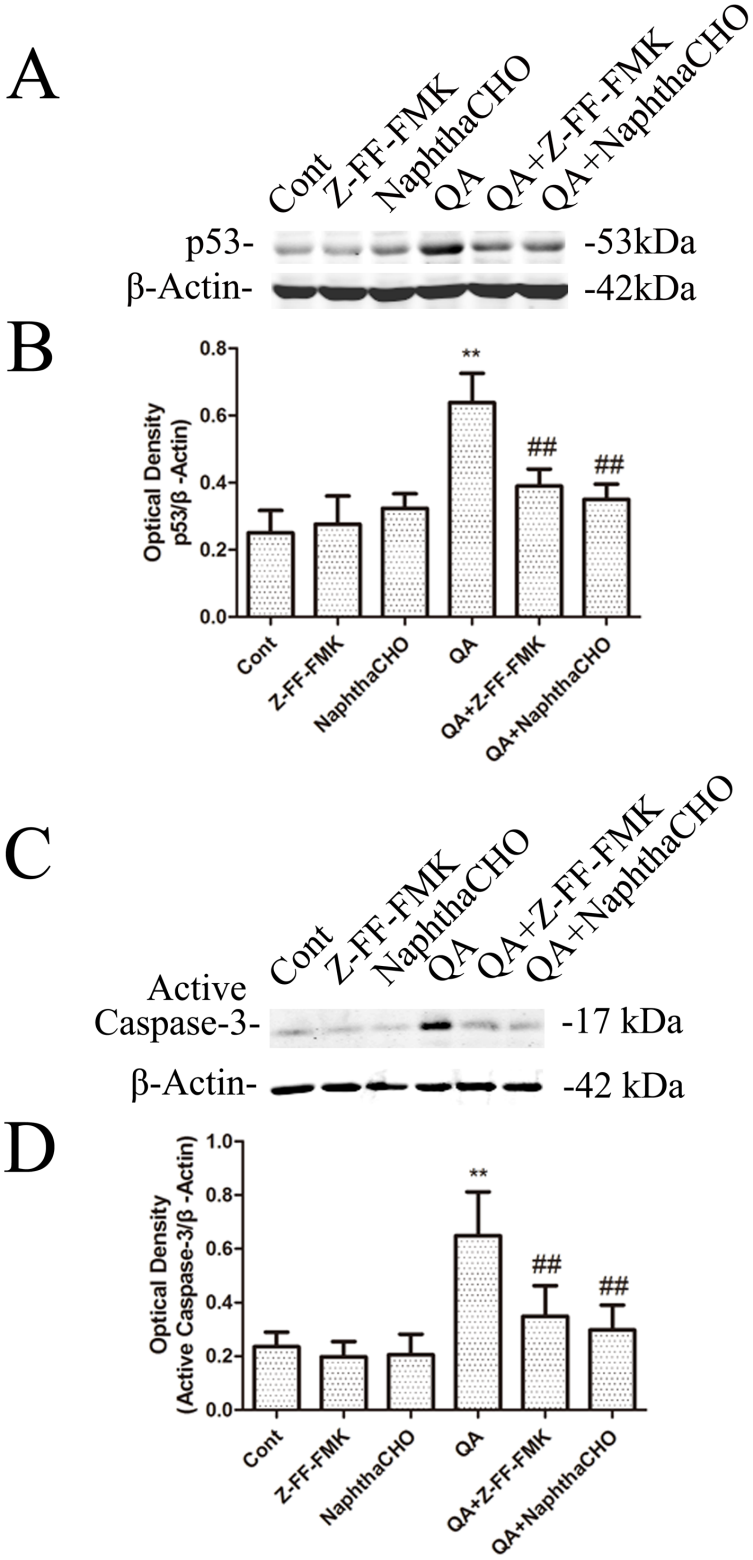
The effects of Z-FF-FMK and NaphthaCHO on QA-induced increases in P53 protein levels and activation of caspase-3. Rats were treated with intrastriatal injection of Z-FF-FMK (5 nmol) or NaphthaCHO (5 nmol) 10 min prior to QA (60 nmol) injection. Rats were killed 12 h later after QA injection. Striatal tissues were dissected for preparation of total lysates. The protein levels of P53 and active caspase-3 were determined with immunoblotting. Bars represent mean ± SEM, n = 6 animals per group. Statistical comparisons were carried out with one-way ANOVA followed by Bonferroni *t*-test. ** *P*<0.01 *vs.* control group; ## *P*<0.01 *vs.* QA-treated group.

NF-κB nuclear translocation mediates the upregulation of TP53 and c-Myc in striatal neurons exposed to excitotoxic injury [32], [33], [35]. Also, QA-induced neuronal apoptosis involves induction of TP53 proapoptotic proteins [9]. In the present study, the effects of cathepsin L inhibitors on pro-apoptotic and autophagy regulatory proteins were assessed to evaluate if cathepsin L plays a regulatory role on QA-induced neuronal apoptotic and autophagic cell death. The results have shown that a significant augmentation in protein levels of TP53 was observed 12 h after intrastriatal infusion of QA, and pretreatment with intrastriatal infusion of the cathepsin L inhibitor Z-FF-FMK or NaphthaCHO resulted in a significant inhibition on QA-induced elevations of TP53 (Figure 6A and 6B) in rat striatum. Apoptosis mediated by TP53 is dependent on p21 and p90 [36], [37], mitochondrial cytochrome c release, and caspase activation [38]. Immunoblotting after QA administration was used in this study to assess the activation of caspase-3. The results have shown that QA induced an increase in active caspase-3 (p17) protein levels. Z-FF-FMK or NaphthaCHO markedly inhibited QA-induced production of active caspase-3 (p17) (Figure 6C and 6D).

**Figure 7:**
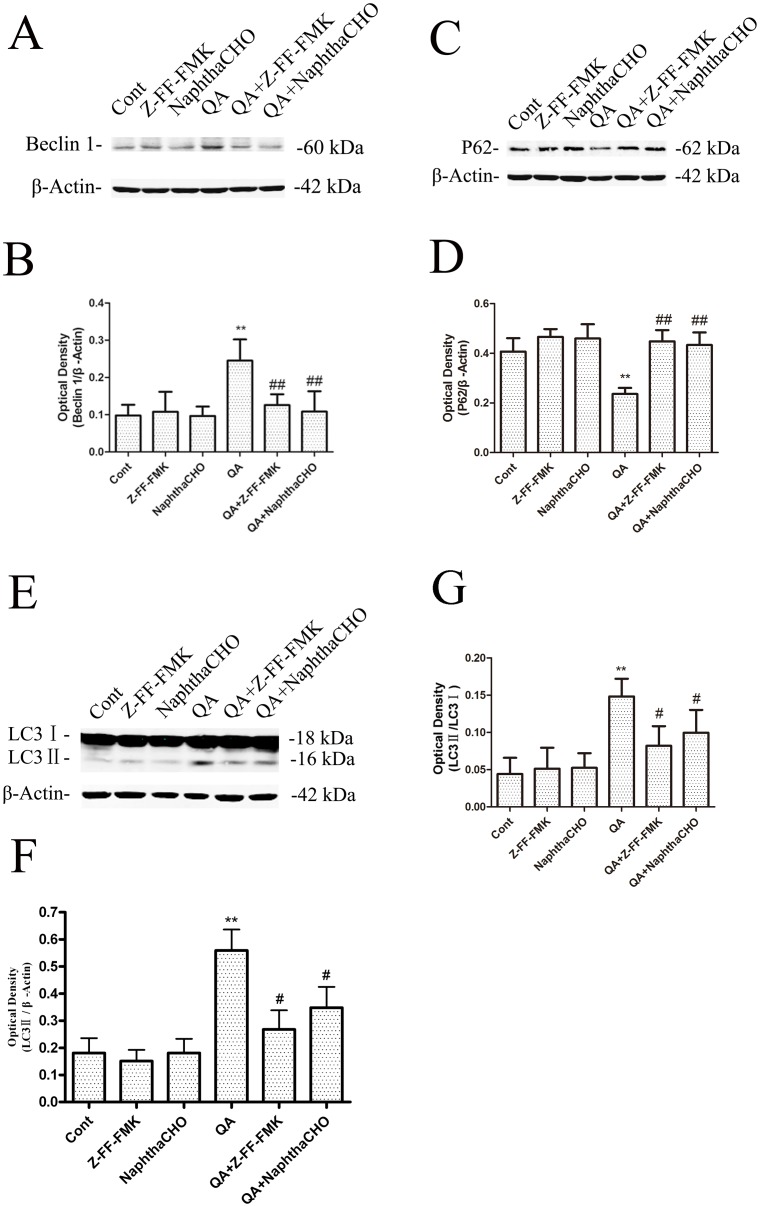
The effects of Z-FF-FMK and NaphthaCHO on QA-induced increase in Beclin 1, LC3II/LC3I and decrease in P62 protein levels. Rats were treated with intrastriatal injection of Z-FF-FMK (5 nmol) or NaphthaCHO (5 nmol) 10 min prior to QA (60 nmol) injection. Rats were killed 12 h later after QA injection. Striatal tissues were dissected for preparation of total lysates. The protein levels of Beclin1, LC3II/LC3I and P62 were determined with immunoblotting. Bars represent mean ± SEM, n = 6 animals per group. Statistical comparisons were carried out with one-way ANOVA followed by Bonferroni *t*-test. * *P*<0.05 *vs.* control group; ** *P*<0.01 *vs.* control group; # *P* < 0.05 *vs.* QA-treated group; ## *P* < 0.01 *vs.* QA-treated group.

Beclin 1 protein is an important regulator of autophagy. Western blot analysis was used to examine expression of beclin 1. The results demonstrated that levels of beclin 1 elevated 12-h after QA infusion and were significantly lowered in rats pretreated with Z-FF-FMK or NaphthaCHO (Figure 7A and 7B). As an LC3-interacting protein that is constantly degraded by autophagy, p62 has been widely used as a marker for autophagic flux [39]. A significant reduction in p62 protein levels was seen 12 h after QA treatment. Pretreatment with Z-FF-FMK or NaphthaCHO significantly reduced the downregulation of p62 (Figure 7C and 7D). LC3-II is required for the formation of autophagosomes and has been defined as a biomarker of autophagosomes in mammalian cells [40]. LC3-II is the cleaved and lipidated form of the cytosolic LC3-I. The protein levels of LC3 were determined with Western blot analysis. The results have shown that LC3II/LC3I was increased 12 h following QA administration and was significantly lowered in rats pretreated with Z-FF-FMK or NaphthaCHO (Figure 7E, 7F and 7G). In contrast, lysosomal inhibitor chloriquine (Figure S4) or cathepsin B inhibitor Ac-LVK-CHO (Figure S5) caused accumulation of LC3-II and P62, but had no additional effects on QA-induced upregulation of LC3II/LC3I, possibly due to a ceiling effect on LC3II/LC3I by QA. These two inhibitors also partially reversed QA-induced downregulation of P62.

## Discussion

Studies have shown that cathepsin L was implicated in Aβ-neurotoxicity in the cortex [41]. Whether cathepsin L contributes to NMDA receptor-induced excitotoxicity is unknown. The present study analyzed the effects of Z-FF-FMK and NaphthaCHO on QA-triggered striatal cell death to evaluate the role of cathepsin L in QA-induced excitotoxic neuronal death. The data demonstrated that Z-FF-FMK and NaphthaCHO significantly reduced QA-induced striatal damage. The results have shown that cathepsin L was involved in QA-induced excitotoxic neuronal death and that inhibition of cathepsin L had neuroprotective effects. However, there was no linear dose-effect relationship for Z-FF-FMK. We observed that 10 nmol Z-FF-FMK caused minor striatal damage. This may be the reason why higher dose of Z-FF-FMK failed to offer greater neuroprotection.

Our previous studies proved that QA-induced neuronal death was accompanied by activation of NF-κB and the inhibition of NF-κB activity blocked QA-induced neuronal injury [42], [43]. To identify if cathepsin L contributes to QA-induced activation of NF-κB, we analyzed the effects of QA on cathepsin L and the effects of Z-FF-FMK and NaphthaCHO on QA-induced IκB-α degradation, phosphorylation, and cellular localization of NF-κB p65. The studies demonstrated that the protein level of cathepsin L and the activity of cathepsin L were increased after QA injection. The cathepsin L inhibitors Z-FF-FMK and NaphthaCHO effectively inhibited cathepsin L protein level and activity. Though we used two kinds of cathepsin L inhibitors with different mechanisms to address the specificity of the cathepsin inhibitors, it is ideal to use cathepsin L knockout animals to more precisely address the involvement of cathepsin L in excitotoxicity. We do not have access to cathepsin L knockout mice at this time, however, they may be used in our future studies.

The cathepsin L inhibitors Z-FF-FMK and NaphthaCHO blocked QA-induced NF-κB p65 nuclear translocation by inhibition of degradation and phosphorylation and degradation of IκB-α in the present study. Previous studies reported that both apoptotic and autophagic mechanisms were involved in excitotoxic cell death. We analyzed the effects of the cathepsin L inhibitors Z-FF-FMK and NaphthaCHO on QA-triggered apoptotic and autophagic activation in order to evaluate if these two cell death pathways are affected by cathepsin L. Our results have shown that Z-FF-FMK and NaphthaCHO not only inhibited QA-induced upregulation of TP53 and other apoptotic proteins such as caspase-3, but also inhibited QA-induced elevation of LC3II/LC3I, beclin1, and downregulation of p62. These data suggested that apoptosis and autophagy activation appeared to depend, at least partially, on cathepsin L. These results were inconsistent with that of other lysosomal inhibitors, such as chloriquine, as they block lysosomal degradation. The inhibition of autophagy activation by cathepsin L can be explained by its effects on NF-κB-TP53 signaling, as this pathway is involved in autophagy activation.

NF-κB regulates the expression of many genes that participate in a variety of physiological responses including apoptosis, neural development, and inflammatory responses [44]–[46]. We have reported that NF-κB activation increased TP53 and c-Myc and promoted neuronal apoptosis. Inhibition of NF-κB nuclear translocation could block these effects [33], [43]. Our recent studies demonstrated that SN50, a NF-κB inhibitor, not only inhibited QA-induced upregulation of TP53 and its target genes involved in signaling apoptosis, but also inhibited QA-induced upregulation of TP53 target gene DRAM1 and other autophagic proteins [9]. In the present study, we observed that cathepsin L inhibitors not only inhibited QA-induced activation of NF-κB, but also inhibited QA-induced upregulation of TP53. TP53 increases the expression of Bax and downregulates Bcl-2 and these could promote cytochrome c release from mitochondria and activate caspase-3 [33], [47], [48]. Caspases are pivotal mediators of apoptosis. Caspase-3 (previously called CPP32, Yama, apopain) is the major downstream protease in all apoptotic pathways [49]. Our studies observed that cathepsin L inhibitors could also reduce QA-induced activation of caspase-3 by inhibition NF-κB nuclear translocation and the expression of its target gene TP53. It is also reported that TP53 is involved in autophagy activation in kainic acid- and 3-nitropropionic acid-induced excitotoxicity [50], [51]. In human hepatocellular carcinoma cells, TP53 is involved in autophagy signaling pathways by fangchinoline [52].

Beclin 1 complex is involved in autophagosome formation at an early stage. This complex is essential for the recruitment of other Atg proteins to the pre-autophagosomal structure [53]. The present studies reveal that QA-induced expression of beclin 1 was inhibited by cathepsin L inhibitors. The ratio of LC3II/LC3I significantly increased after QA treatment. This phenomenon was prevented by cathepsin L inhibitors. When autophagy is induced, LC3-I is modified by the ubiquitin-like conjugation system using Atg7 and Atg3 as E1 and E2 enzymes to add phosphatidylethanolamine at its carboxyl terminal glycine residue, and becomes membrane-bound LC3-II [54], [55]. Because it is bound to the autophagosomal membrane, LC3-II is a marker for autophagy [40]. However, LC3-II can accumulate when maturation of autophagy is compromised [56], so we detected the level of autophagy by another autophagy related protein, p62. The adaptor protein p62 is an autophagy-targeting molecule recognizing ubiquitinated cytoplasmic components and delivering them for degradation [57]–[62]. In the present studies, we find that downregulation of p62 induced by QA was inhibited when pretreated with cathepsin L inhibitors. Our studies have shown that cathepsin L inhibitors could also reduce QA-induced activation of autophagy by inhibiting NF-κB nuclear translocation and the expression of its target gene p53. However, it was reported that TP53 regulated the permeabilization of the lysosomal membrane to some extent, which induced the leakage of lysosomal enzymes and downregulation of p62 [63], [64]. Pan, *et al* reported that protein aggregation upon the inhibition of proteasomal and autophagic degradation pathways was mediated by the ubiquitin binding protein SQSTM1/p62 and the autophagy-related protein LC3. Also, silencing of p62 and LC3 protects cells from MDEG G430F-induced cell death [65]. In the present studies, the protein expression changes of beclin 1, LC3II/LC3I, and p62 show the activation of autophagy. We also used chloriquine, which could disrupt the lysosomal activity [66], in our experiments. The results show that chloriquine increased LC3II/LC3I levels but had no additional effects on QA-induced increases in LC3II/LC3I. Cathepsin B inhibitor Ac-LVK-CHO significantly reduced QA-induced striatal cell death and caused accumulation of LC3-II, but had no additional effects on QA-induced upregulation of LC3II/LC3I, possibly due to a ceiling effect on LC3II/LC3I by QA.

The present data demonstrate that cathepsin L is involved in NMDA receptor-mediated IκB-α degradation and NF-κB activation. Inhibition of cathepsin L attenuates both autophagic and apoptotic pathways after excitotoxic exposure (Figure S6). These studies suggest that cathepsin L plays an important role in excitotoxicity. Therefore, this study may provide new tactics for the treatment of neurodegenerative diseases.

## Supporting Information

Figure S1 **The effects of Z-FF-FMK (10 nmol) on striatal neurons.** Rats were treated with intrastriatal injection of Z-FF-FMK (10 nmol). Rats were killed 14 days after treatment. Paraformaldehyde-fixed brain sections were stained with Nissl. **A:** The effects of Z-FF-FMK under control conditions. Representative micrographs were taken in the center of drug injection (adjacent to needle tracks). **a and b:** Vehicle. **c and d:** Z-FF-FMK (10 nmol). b and d (×200) were enlarged from areas indicated with asterisks in a and c (×20). Scale bar  = 200 µm in (a and c);  = 20 µm in (b and d). **B:** Quantitative analysis of the effects of Z-FF-FMK (10 nmol) on lesion size under control conditions. Three Nissl-stained sections from each animal were used for quantitative analysis of lesion size caused by Z-FF-FMK. Plate 1: sections taken at 1.4 mm anterior to the Bregma; Plate 2: sections taken at 0.6 mm anterior to the Bregma; Plate 3: sections taken at 0.2 mm posterior to the Bregma (The Stereotaxic Atlas of The Rat Brain by Xin-Min Bao, Si-Yun Shu; People's Health Press). Striatal images were captured and exported to Sigma Scan Pro 5 for determining lesion size. Lesion size was expressed as percent of the total size of the striatum of each section. Bars represent mean ± SEM, n = 6 animals per group. Statistical comparisons were carried out with one-way ANOVA followed by Bonferroni *t* test. **C:** The effects of Z-FF-FMK on loss of striatal neurons under control conditions. 12 Nissl-stained sections (with the interval of every 14 successive brain sections) were used for counting neuronal numbers with an Optical Fractionator microscopy and stereology software. The neuronal number of the total striatum was expressed as percent of control (vehicle-treated group). Bars represent mean ± SEM, n = 6 animals per group. Statistical comparisons were carried out with one-way ANOVA followed by Bonferroni *t* test. The difference was not statistically significant between vehicle and Z-FF-FMK (10 nmol) treatment.
(TIF)

Figure S2 **The effects of the cathepsin B inhibitor Ac-LVK-CHO on QA-induced striatal damage.** Rats were treated with intrastriatal injection of Ac-LVK-CHO (10 nmol) 10 min prior to QA (60 nmol) injection. Rats were killed 14 days after QA treatment. Paraformaldehyde-fixed brain sections were stained with Nissl. **A:** The effects of Ac-LVK-CHO on QA-induced striatal damage. Representative micrographs were taken in the center of drug injection (adjacent to needle tracks). a and b: Vehicle. c and d: Ac-LVK-CHO (10 nmol). e and f: QA. g and h: QA+Ac-LVK-CHO (10 nmol). b, d, f and h (×200) were enlarged from areas indicated with asterisks in a, c, e and g (×20). Scale bar  = 200 µm in (a, c, e and g);  = 20 µm in (b, d, f and h). **B:** Quantitative analysis of the effects of Ac-LVK-CHO (10 nmol) on lesion size. Three Nissl-stained sections from each animal were used for quantitative analysis of lesion size caused by QA. Plate 1: sections taken at 1.4 mm anterior to the Bregma; Plate 2: sections taken at 0.6 mm anterior to the Bregma; Plate 3: sections taken at 0.2 mm posterior to the Bregma (The Stereotaxic Atlas of The Rat Brain by Xin-Min Bao, Si-Yun Shu; People's Health Press). Striatal images were captured and exported to Sigma Scan Pro 5 for determining lesion size. Lesion size was expressed as percent of the total size of the striatum of each section. Bars represent mean ± SEM, n = 6 animals per group. Statistical comparisons were carried out with one-way ANOVA followed by Bonferroni *t* test. **C:** The effects of Ac-LVK-CHO on QA-induced loss of striatal neurons. 12 Nissl-stained sections (with the interval of every 14 successive brain sections) were used for counting neuronal numbers with an Optical Fractionator microscopy and stereology software. The neuronal number of the total striatum was expressed as percent of control (vehicle-treated group). Bars represent mean ± SEM, n = 6 animals per group. Statistical comparisons were carried out with one-way ANOVA followed by Bonferroni *t* test. The difference was not statistically significant between vehicle and Ac-LVK-CHO treatment. ** *P*<0.01 *vs.* control group; ## *P*<0.01 *vs.* QA-treated group.
(TIF)

Figure S3 **Effects of NaphthaCHO on cathepsin B activation.** Rats were treated with intrastriatal injection of NaphthaCHO (5 nmol) and were killed 12 h later. Control animals received vehicle injection only. **A:** Striata were dissected for assay of cathepsin B activity using a fluorescence-based assay kit. The results were expressed as percent of control (vehicle injection) after performing statistical analysis. **B and C:** Striata were dissected for assay of cathepsin B protein level using Western Blot. Bars represent Mean ± SEM (n = 6). Statistical comparisons were carried out with one-way ANOVA followed by Bonferroni *t*-test. The difference was not statistically significant between vehicle and NaphthaCHO (5 nmol) treatment.
(TIF)

Figure S4 **The effects of Chloriquine on QA-induced upregulation of LC3II/LC3I and downregulation of P62.** Rats were treated with intrastriatal injection of Chloriquine (100 nmol) 10 min prior to QA (60 nmol) injection. Rats were killed 12 h later after QA injection. Striatal tissues were dissected for preparation of total lysates. The protein levels of LC3II/LC3I and P62 were determined with immunoblotting. Bars represent mean ± SEM, n = 6 animals per group. Statistical comparisons were carried out with one-way ANOVA followed by Bonferroni *t*-test. The difference was not statistically significant between QA and QA+CQ treatment. * *P*<0.05 *vs.* control group; ** *P*<0.01 *vs.* control group.
(TIF)

Figure S5 **The effects of Ac-LVK-CHO on QA-induced upregulation of LC3II/LC3I and downregulation of P62.** Rats were treated with intrastriatal injection of Ac-LVK-CHO (10 nmol) 10 min prior to QA (60 nmol) injection. Rats were killed 12 h later after QA injection. Striatal tissues were dissected for preparation of total lysates. The protein levels of LC3II/LC3I and P62 were determined with immunoblotting. Bars represent mean ± SEM, n = 6 animals per group. Statistical comparisons were carried out with one-way ANOVA followed by Bonferroni *t*-test. * *P*<0.05 *vs.* control group.
(TIF)

Figure S6 **The mechanistic pathway to explain cathepsin L involvement in NF-κB activation, as well as autophagy/lysosomal pathway in QA-induced neuronal cell death.**
(TIF)
