# Supplementary figures and images for: Cathepsin L Plays a Role in Quinolinic Acid-Induced NF-Κb Activation and Excitotoxicity in Rat Striatal Neurons
Source: PLoS One. 2013 Sep 20;8(9):e75702. doi: 10.1371/journal.pone.0075702 (PMC3779166; doi:10.1371/journal.pone.0075702)

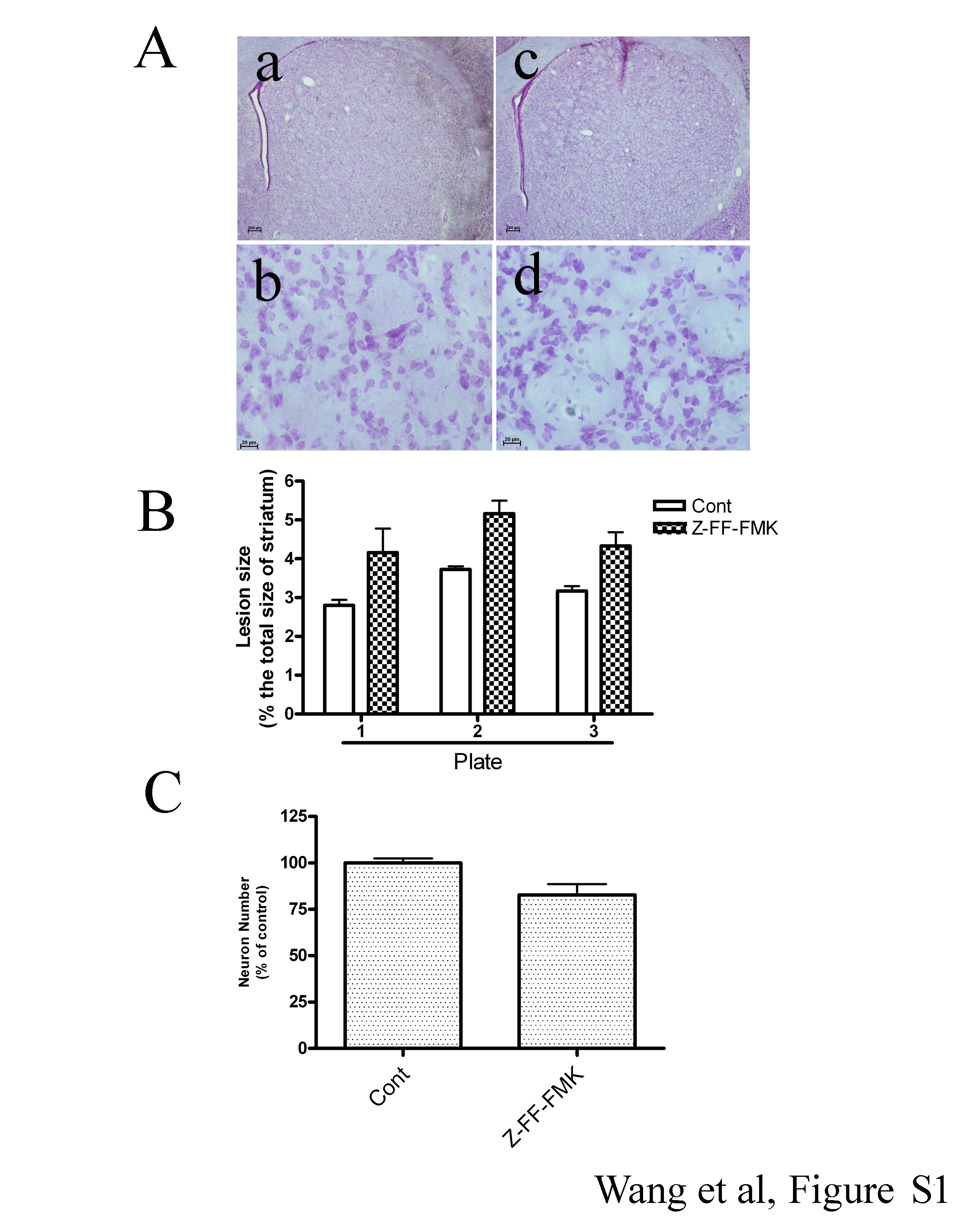

Supplement: Figure S1 — The effects of Z-FF-FMK (10 nmol) on striatal neurons. Rats were treated with intrastriatal injection of Z-FF-FMK (10 nmol). Rats were killed 14 days after treatment. Paraformaldehyde-fixed brain sections were stained with Nissl. A: The effects of Z-FF-FMK under control conditions. Representative micrographs were taken in the center of drug injection (adjacent to needle tracks). a and b: Vehicle. c and d: Z-FF-FMK (10 nmol). b and d (×200) were enlarged from areas indicated with asterisks in a and c (×20). Scale bar = 200 µm in (a and c); = 20 µm in (b and d). B: Quantitative analysis of the effects of Z-FF-FMK (10 nmol) on lesion size under control conditions. Three Nissl-stained sections from each animal were used for quantitative analysis of lesion size caused by Z-FF-FMK. Plate 1: sections taken at 1.4 mm anterior to the Bregma; Plate 2: sections taken at 0.6 mm anterior to the Bregma; Plate 3: sections taken at 0.2 mm posterior to the Bregma (The Stereotaxic Atlas of The Rat Brain by Xin-Min Bao, Si-Yun Shu; People's Health Press). Striatal images were captured and exported to Sigma Scan Pro 5 for determining lesion size. Lesion size was expressed as percent of the total size of the striatum of each section. Bars represent mean ± SEM, n = 6 animals per group. Statistical comparisons were carried out with one-way ANOVA followed by Bonferroni t test. C: The effects of Z-FF-FMK on loss of striatal neurons under control conditions. 12 Nissl-stained sections (with the interval of every 14 successive brain sections) were used for counting neuronal numbers with an Optical Fractionator microscopy and stereology software. The neuronal number of the total striatum was expressed as percent of control (vehicle-treated group). Bars represent mean ± SEM, n = 6 animals per group. Statistical comparisons were carried out with one-way ANOVA followed by Bonferroni t test. The difference was not statistically significant between vehicle and Z-FF-FMK (10 nmol) treatment. (TIF) [file pone.0075702.s001.tif]

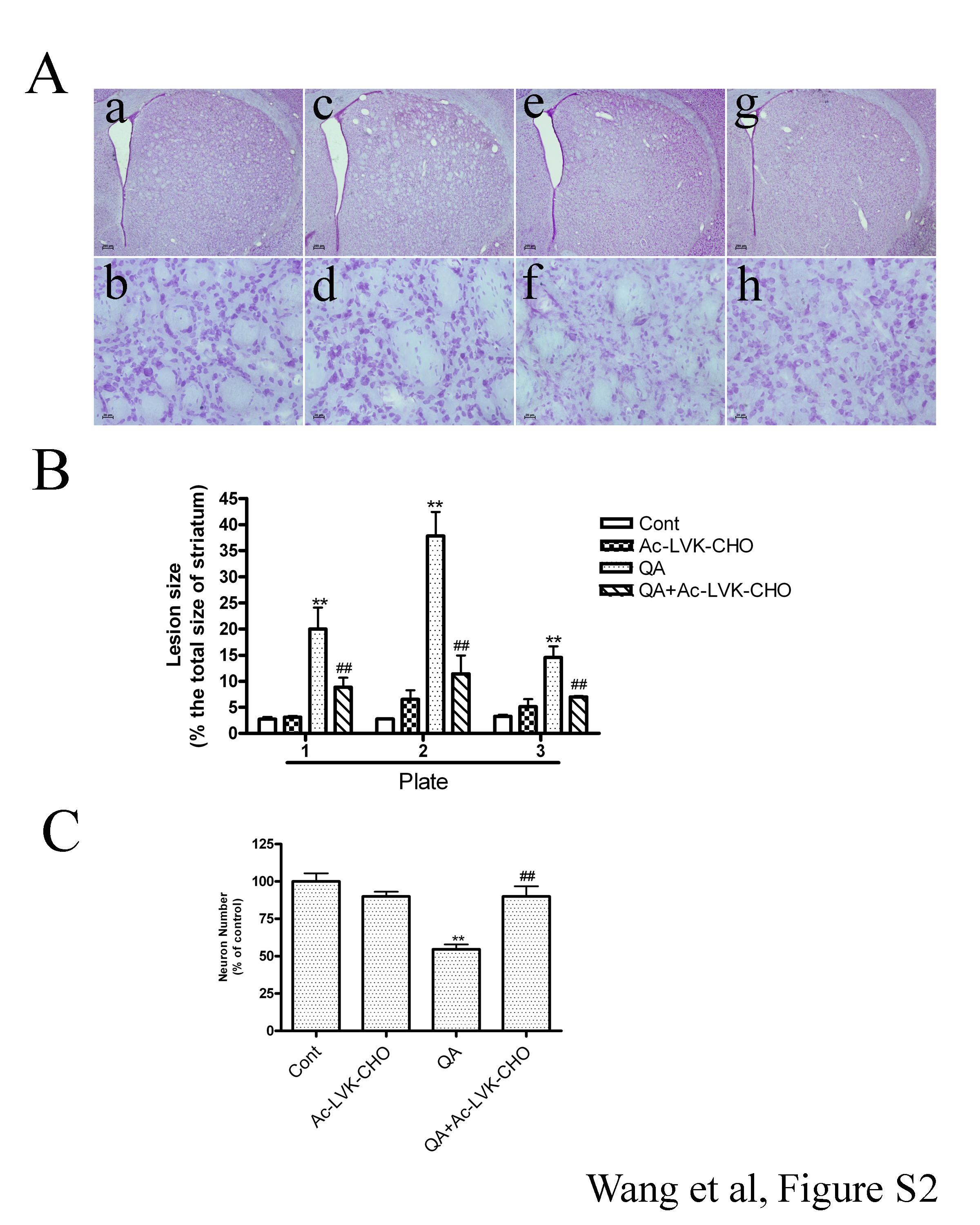

Supplement: Figure S2 — The effects of the cathepsin B inhibitor Ac-LVK-CHO on QA-induced striatal damage. Rats were treated with intrastriatal injection of Ac-LVK-CHO (10 nmol) 10 min prior to QA (60 nmol) injection. Rats were killed 14 days after QA treatment. Paraformaldehyde-fixed brain sections were stained with Nissl. A: The effects of Ac-LVK-CHO on QA-induced striatal damage. Representative micrographs were taken in the center of drug injection (adjacent to needle tracks). a and b: Vehicle. c and d: Ac-LVK-CHO (10 nmol). e and f: QA. g and h: QA+Ac-LVK-CHO (10 nmol). b, d, f and h (×200) were enlarged from areas indicated with asterisks in a, c, e and g (×20). Scale bar = 200 µm in (a, c, e and g); = 20 µm in (b, d, f and h). B: Quantitative analysis of the effects of Ac-LVK-CHO (10 nmol) on lesion size. Three Nissl-stained sections from each animal were used for quantitative analysis of lesion size caused by QA. Plate 1: sections taken at 1.4 mm anterior to the Bregma; Plate 2: sections taken at 0.6 mm anterior to the Bregma; Plate 3: sections taken at 0.2 mm posterior to the Bregma (The Stereotaxic Atlas of The Rat Brain by Xin-Min Bao, Si-Yun Shu; People's Health Press). Striatal images were captured and exported to Sigma Scan Pro 5 for determining lesion size. Lesion size was expressed as percent of the total size of the striatum of each section. Bars represent mean ± SEM, n = 6 animals per group. Statistical comparisons were carried out with one-way ANOVA followed by Bonferroni t test. C: The effects of Ac-LVK-CHO on QA-induced loss of striatal neurons. 12 Nissl-stained sections (with the interval of every 14 successive brain sections) were used for counting neuronal numbers with an Optical Fractionator microscopy and stereology software. The neuronal number of the total striatum was expressed as percent of control (vehicle-treated group). Bars represent mean ± SEM, n = 6 animals per group. Statistical comparisons were carried out with one-way ANOVA followed by Bonferroni t tes [file pone.0075702.s002.tif]

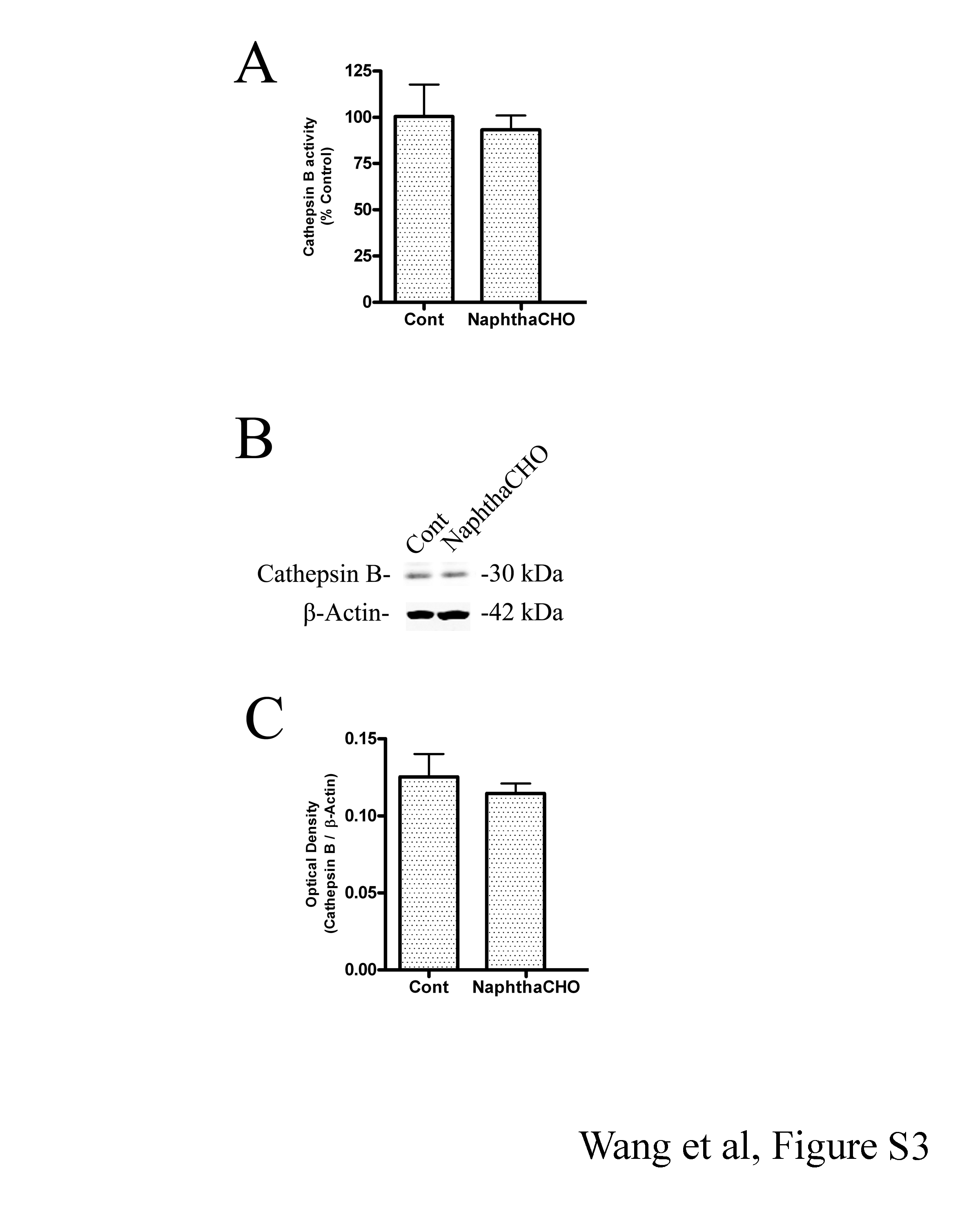

Supplement: Figure S3 — Effects of NaphthaCHO on cathepsin B activation. Rats were treated with intrastriatal injection of NaphthaCHO (5 nmol) and were killed 12 h later. Control animals received vehicle injection only. A: Striata were dissected for assay of cathepsin B activity using a fluorescence-based assay kit. The results were expressed as percent of control (vehicle injection) after performing statistical analysis. B and C: Striata were dissected for assay of cathepsin B protein level using Western Blot. Bars represent Mean ± SEM (n = 6). Statistical comparisons were carried out with one-way ANOVA followed by Bonferroni t-test. The difference was not statistically significant between vehicle and NaphthaCHO (5 nmol) treatment. (TIF) [file pone.0075702.s003.tif]

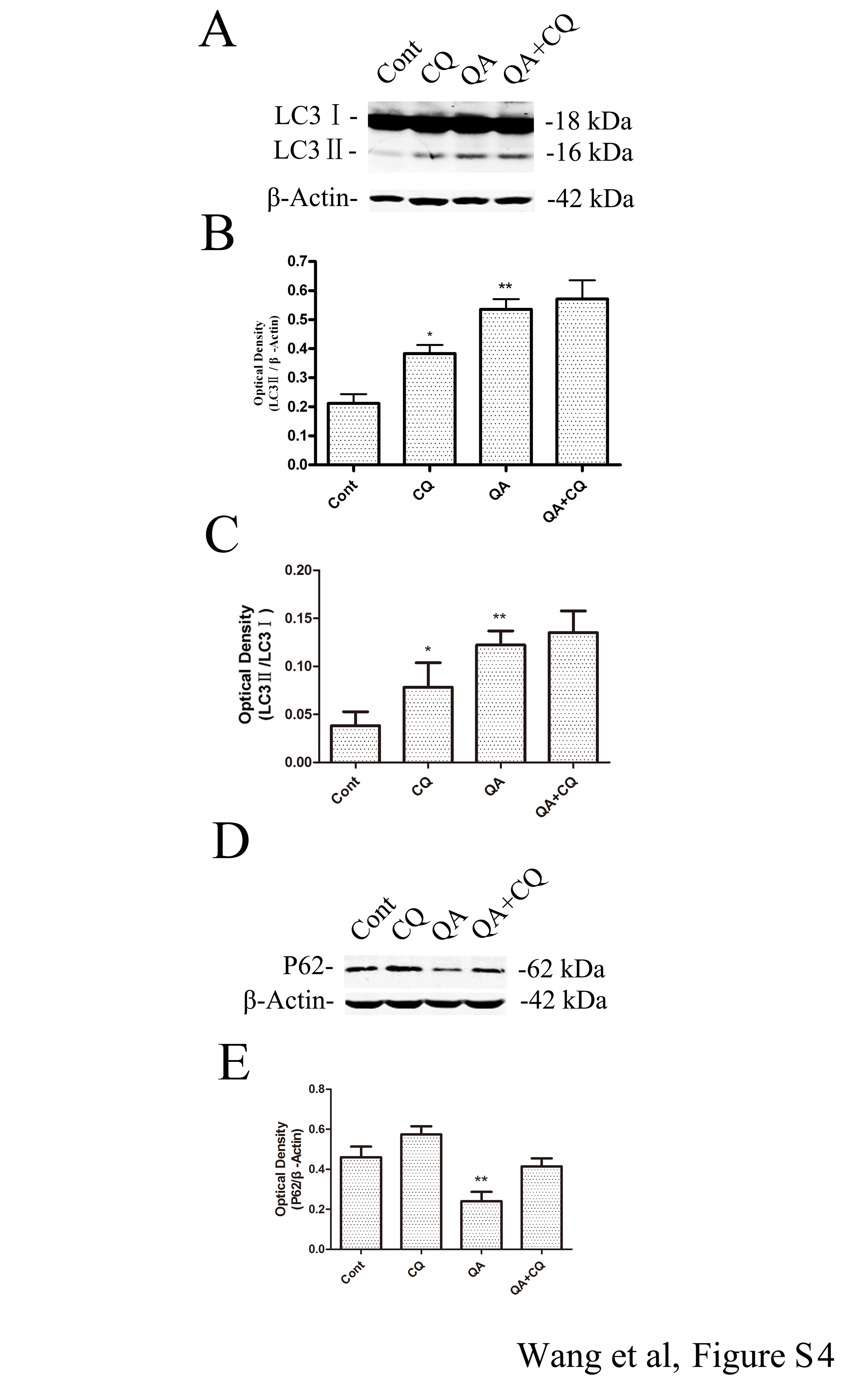

Supplement: Figure S4 — The effects of Chloriquine on QA-induced upregulation of LC3II/LC3I and downregulation of P62. Rats were treated with intrastriatal injection of Chloriquine (100 nmol) 10 min prior to QA (60 nmol) injection. Rats were killed 12 h later after QA injection. Striatal tissues were dissected for preparation of total lysates. The protein levels of LC3II/LC3I and P62 were determined with immunoblotting. Bars represent mean ± SEM, n = 6 animals per group. Statistical comparisons were carried out with one-way ANOVA followed by Bonferroni t-test. The difference was not statistically significant between QA and QA+CQ treatment. * P<0.05 vs. control group; ** P<0.01 vs. control group. (TIF) [file pone.0075702.s004.tif]

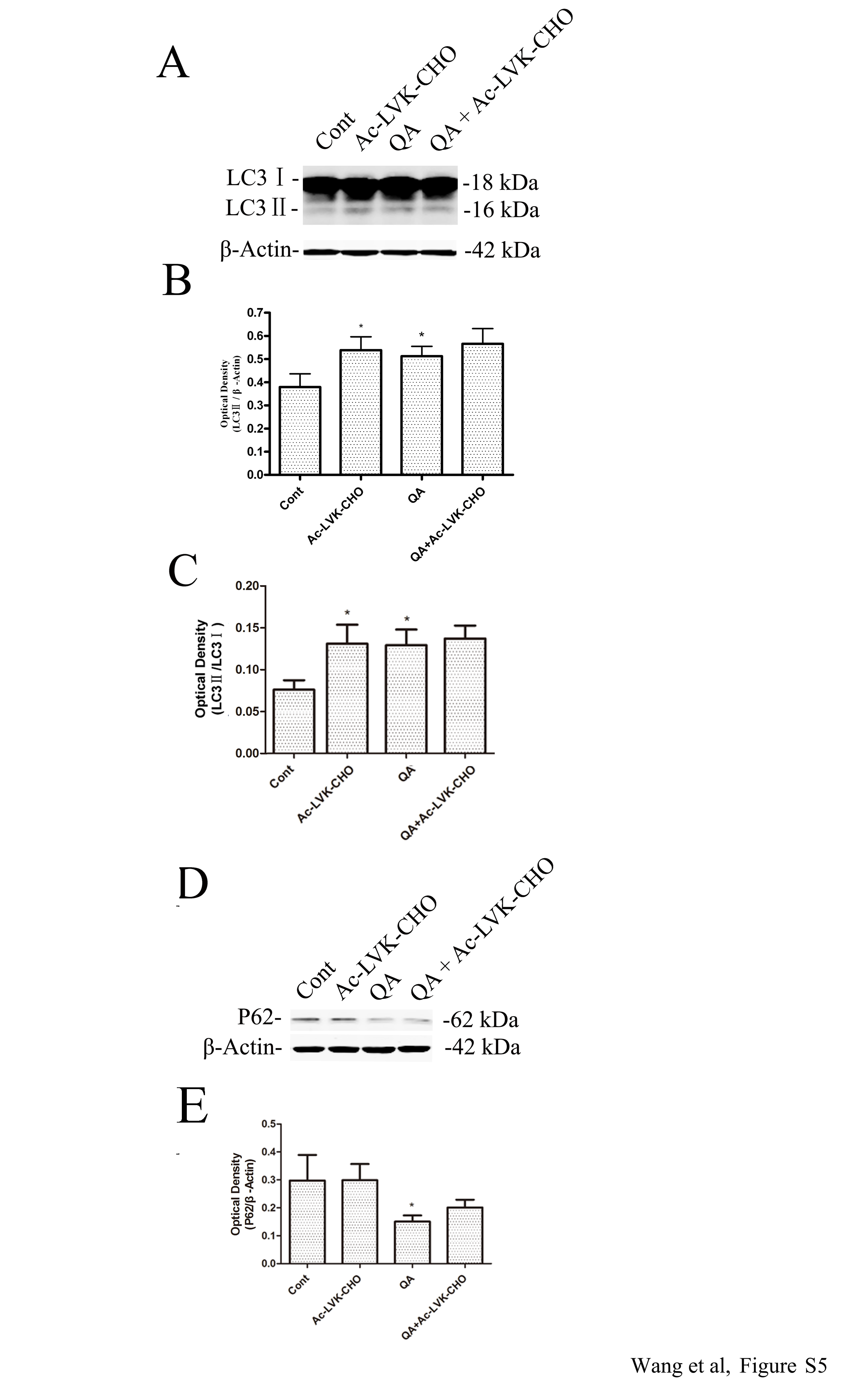

Supplement: Figure S5 — The effects of Ac-LVK-CHO on QA-induced upregulation of LC3II/LC3I and downregulation of P62. Rats were treated with intrastriatal injection of Ac-LVK-CHO (10 nmol) 10 min prior to QA (60 nmol) injection. Rats were killed 12 h later after QA injection. Striatal tissues were dissected for preparation of total lysates. The protein levels of LC3II/LC3I and P62 were determined with immunoblotting. Bars represent mean ± SEM, n = 6 animals per group. Statistical comparisons were carried out with one-way ANOVA followed by Bonferroni t-test. * P<0.05 vs. control group. (TIF) [file pone.0075702.s005.tif]

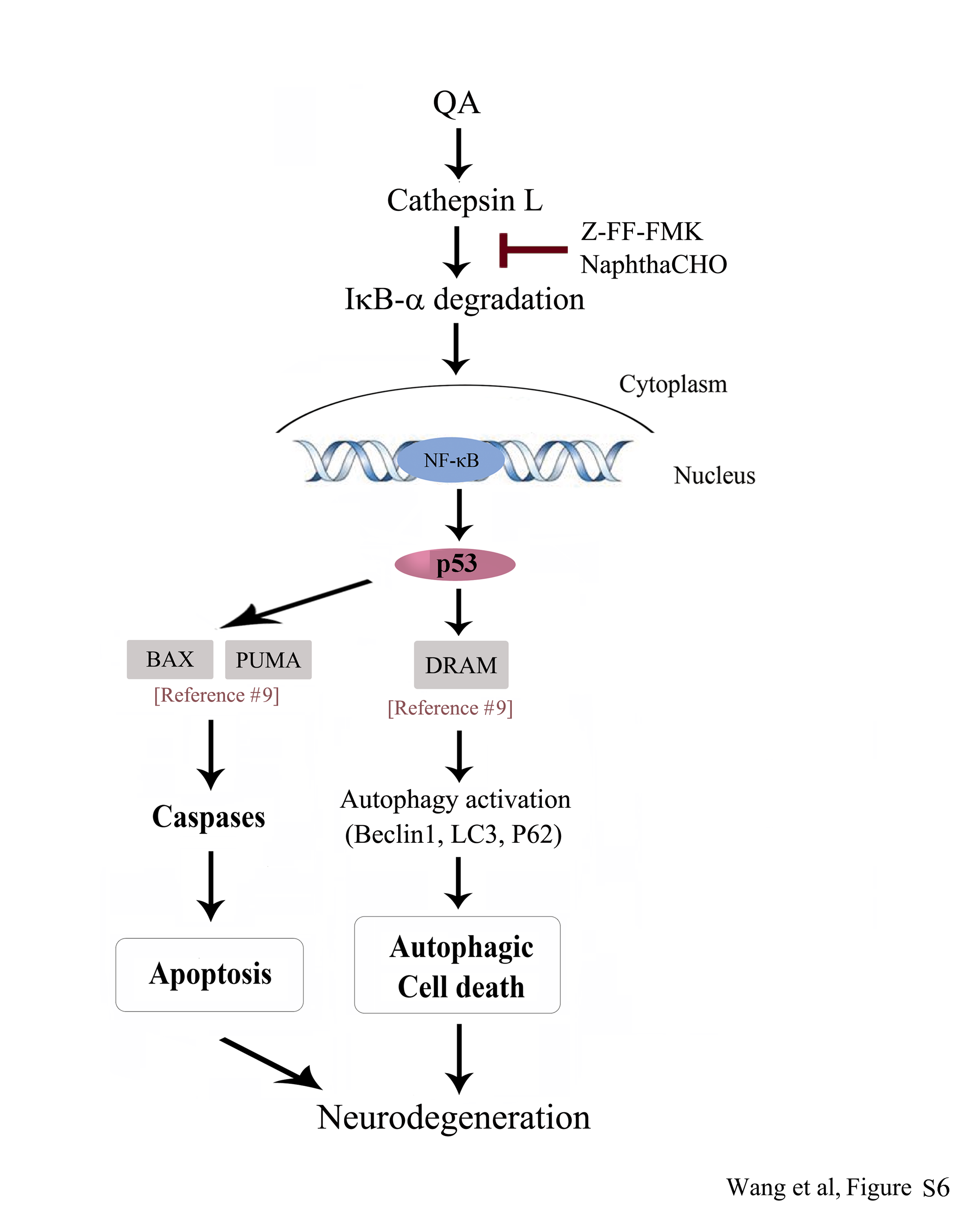

Supplement: Figure S6 — The mechanistic pathway to explain cathepsin L involvement in NF-κB activation, as well as autophagy/lysosomal pathway in QA-induced neuronal cell death. (TIF) [file pone.0075702.s006.tif]
